# Exploratory Study: Excessive Iron Supplementation Reduces Zinc Content in Pork without Affecting Iron and Copper

**DOI:** 10.3390/ani11030776

**Published:** 2021-03-11

**Authors:** Maureen Middleton, Manuel Olivares, Alejandra Espinoza, Miguel Arredondo, Fernando Pizarro, Carolina Valenzuela

**Affiliations:** 1 Laboratorio de Micronutrientes, Instituto de Nutrición y Tecnología de los Alimentos (INTA), Universidad de Chile, Av El Libano 5524, Macul, Santiago 7830490, Chile; maureen.middleton.s@gmail.com (M.M.); molivare@inta.uchile.cl (M.O.); marredon@inta.uchile.cl (M.A.); 2 Escuela de Ingeniería Civil Industrial, Universidad Diego Portales, Vergara 432, Santiago 8370014, Chile; alejandra.espinoza@udp.cl; 3 Facultad de Ciencias Veterinarias y Pecuarias, Universidad de Chile, Av. Santa Rosa 11735, La Pintana, Santiago 8820808, Chile

**Keywords:** copper, iron, supplementation, pigs, pork, offal, zinc

## Abstract

**Simple Summary:**

Currently, all pigs raised on intensive farms develop iron-deficiency anemia if they do not receive supplemental iron at birth. Weaning diets commonly contain high concentrations of iron, and the effect on the copper and zinc contents in pork is unknown. In this exploratory work, we determined the effect of excessive oral iron supplementation on the contents of these microminerals in pork. Surprisingly, we found that high iron doses of 3000 ppm reduced the zinc content of pork by 32–55%.

**Abstract:**

The aim of this work was to determine in an exploratory manner the effect of excessive iron supplementation on iron, zinc, and copper contents in pork and pork offal. Pigs averaging 50 days in age and 15 ± 1.3 kg body weight were allocated to a control group (500 ppm dietary Fe) and a supplemental group (3000 ppm dietary Fe). After an iron supplementation period of 60 days, blood samples were analyzed to determine iron biomarkers, serum copper, and zinc contents. Animals were slaughtered to assess total iron, non-heme iron, heme iron, zinc, and copper contents in samples of nine meat cuts and some offal. Iron supplementation improved the iron status in pigs with increased hemoglobin and hematocrit, but did not affect serum levels of iron, zinc, and copper. Iron supplementation did not affect the heme and non-heme iron contents of the different meat cuts. Zinc contents decreased by 32–55% in meat cuts, where iron content increased in the liver, spleen, kidneys, and pancreas. No differences of zinc and copper were observed in offal samples. High concentrations of iron supplementation reduce zinc content in pork.

## 1. Introduction

Iron, zinc, and copper are trace minerals that play a key role in several biological processes. These microminerals are found mainly in foods of animal origin (e.g., meats and shellfish). Meat and shellfish are rarely consumed in developing countries, resulting in major nutritional deficiencies [1,2,3]. Iron deficiency is the most prevalent nutritional disorder, affecting up to 30% of the world’s population [4]. Iron deficiency can be treated with increased meat consumption [5]. Meat products provide several essential nutrients including proteins, lipids, minerals, and micronutrients, and 50–60% of the total iron present in pork is heme iron, which is a highly bioavailable form of iron [6]. Zinc found in meat is absorbed better than zinc present in vegetables [7].

Mineral supplementation in pigs has been widely researched, especially iron, because of the high prevalence of iron-deficiency anemia that develops between the first and second week of age in pigs raised under intensive conditions. The iron deficiency becomes more acute three weeks after weaning, where the prevalence of anemia (6–32%) and iron deficiency (29–74%) increases [8]. This condition results in low live weights and daily weight gain, impaired functioning of the immune system [9], and greater susceptibility to diseases [10]. Different iron supplementation strategies have been investigated in suckling and weaned piglets [11,12,13]. However, an excess of iron can be harmful to pig health, as it facilitates free radical formation [14], increases the incidence of diarrhea [15], alters iron homeostasis, increases hepcidin to critical levels [16], and causes cognitive impairments [17].

The effect that excessive iron supplementation can have on other microminerals in meat has been poorly studied. Doses of iron greater than 1000 ppm in a pig’s diet are considered high and increase the destruction of tocopherols [18]. However, a dose of 3000 ppm does not affect the performance of pigs. At a dose of 4000 ppm, the growth rate and inorganic phosphorus in serum are reduced. With 5000 ppm, the ash content of the femur is also diminished [19,20]. A dose of 3000 ppm is the maximum tolerable concentration that does not diminish animal performance [21]. For these reasons, we used 3000 ppm of iron in this study. We hypothesized that excessive oral supplementation with 3000 ppm iron could influence the deposit of other minerals in pork, such as zinc and copper, which are absorbed through similar pathways in the intestine. The aim of this work was to determine the effect of excessive iron supplementation on iron, zinc, and copper contents in pork and pork offal.

## 2. Materials and Methods

### 2.1. Animals

Eight commercial hybrid male pigs (TEMPO×TOPIG 20) were used, averaging 50 days in age and 15 ± 1.3 kg body weight. All protocols were approved by the Bioethics Committee from the Institute of Nutrition and Food Technology (INTA) at the University of Chile (Certificate of Bioethics 501-11, project FONDECYT # 1095038). For the calculation of the sample size, an error of α = 0.05, a power of 80%, a difference between the control group and the supplemental group of 0.8 mg iron/100 g of the loin cut, and a standard deviation of 0.3 were considered. The calculated *N* was 4 animals in each group.

### 2.2. Experimental Design

Animals were randomly assigned to one of two groups: (1) the control group (Fe500, *n* = 4), where pigs were fed a standard diet based on NRC recommendations [22] (Table 1) that contained 500 ppm of iron; or (2) the supplemented group (Fe3000, *n* = 4), where pigs were fed the same diet described above supplemented with ferrous sulfate (Veterquímica S.A, Santiago, Chile) with up to a total iron content of 3000 ppm. Food intake was controlled: in the first 20 days, each animal received 700 g/day; in the following 20 days, they received 1 kg/day; and in the last 20 days, they received 1.5 kg/day. All meals were divided into two rations per day.

The proximate composition of the diets was analyzed according to the methodology proposed by the Association of Official Analytical Chemists [23] for moisture (method 945.15), crude protein (Kjeldahl method 945.18, N × 6.25), ether extract (method 945.16), crude fiber (method 962.09), and ash (method 920.153). Meanwhile, iron, copper, and zinc contents were determined following the directives for the acid digestion method proposed by AOAC (1996). Mineral concentrations were measured at specific wavelengths for each element (iron: 248.3, copper: 324.7, and zinc: 213.9) using an atomic absorption spectrophotometer with graphite furnace (SIMAA 6100, PerkinElmer, Waltham, MA, USA).

Each pig was weighed individually on days 0 and 60. The mean weight gain at the end of experimental period of 60 days was calculated.

### 2.3. Hematological Parameters and Micromineral Status

To assess mineral status, 30 mL blood samples were drawn from piglets by jugular venepuncture at days 0 and 60. Total blood was used to determine the following biomarkers of iron nutrition status: hemoglobin, hematocrit, and mean corpuscular volume (Cell-Dyn 3200; Abbott Laboratories, Abbott Park, IL, USA). Serum was separated by centrifugation of whole blood at 1200 × *g* for 3 min, and then immediately stored at −20 °C. Total iron binding capacity (TIBC) was determined in the serum by a colorimetric method [24]. Serum iron, zinc, and copper concentrations were determined by atomic absorption spectrophotometry (SIMAA 6100, PerkinElmer, Waltham, MA, USA). The transferrin saturation percentage (Sat %) was determined using the following formula: Sat (%) = ((Serum iron/TIBC) × 100).

### 2.4. Content of Iron, Copper, and Zinc in Pork and Offal

**Figure 1:**
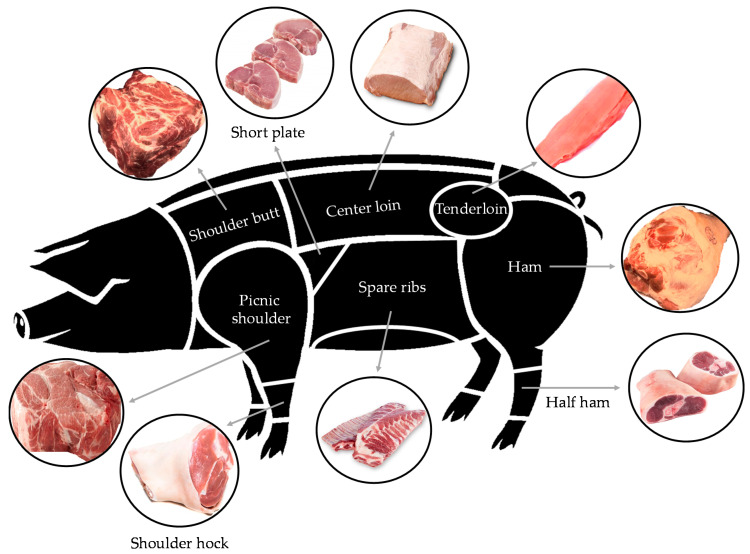
Diagram of American pork cuts (https://animalscience.unl.edu/pork-meat-identification, accessed on 1 February 2021).

Pigs were slaughtered by exsanguination on day 60 after being rendered unconscious using an anesthesia protocol, including an intramuscular injection of 2 mg/kg of azaperone and 5 mg/kg of ketamine. Then, a professional butcher identified and dissected the main pork cuts (Figure 1 and Table 2), which were deboned. From each meat cut (2 samples per hemicanal from the same animal), random samples of each muscle group measuring 1 × 1 × 1 cm^3^ were selected and then frozen at −18 °C. In addition, the liver, spleen, kidneys, heart, brain, and pancreas were collected. Iron, copper, and zinc contents were determined in meat and offal by atomic absorption spectrophotometry after acid digestion [23]. Non-heme iron content was determined by a microanalysis method of non-heme iron in animal tissues [25]. Briefly, tissues were homogenized in deionized water at 1:10 *w/v*. Then, tissue homogenates and protein precipitation solution (1 N HCl and 10% trichloroacetic acid) were combined in micro-centrifuge tubes and placed in a 95 °C heating block for 1 h. The tubes and their contents were cooled in water at room temperature for 2 min, vortexed, and then centrifuged at 16,000 × *g* for 10 min. Supernatant aliquots were mixed with chromogen solution (0.508 mmol/L ferrozine, 1.5 mol/L sodium acetate, and thioglycolic acid at 1.5% *v/v* in deionized water). After 30 min at room temperature, absorbance was measured with a spectrophotometer (SIMAA 6100, PerkinElmer, Waltham, MA, USA). Heme iron content was calculated as the difference of total iron and non-heme iron.

### 2.5. Statistical Analysis

The normality of the data was confirmed by a Shapiro–Wilk test (*p* > 0.05). Pre- and post-supplementation changes of hematological biomarkers and serum microminerals were analyzed with a two-way ANOVA for repeated measures using the GLM procedure of SAS (version 9.0; SAS Institute Inc., Cary, NC, USA). Mean values are presented as least square means adjusted by Tukey’s test. The α level used for the determination of significance was 0.05. The following mathematical model was used (1):

Yijk = µ + αi + βj + γk + εijk(1)

where *Y* is the hematological biomarkers or serum microminerals, µ is the general mean of all observations, *α* is the effect of treatment (control or supplemental group), *β* is the effect of the initial or final time of evaluations (0 and 60 days), *γ* is the interaction (treatment x time), and *ε* is the random error.

Iron, zinc, and copper concentrations in pork samples were compared by a Student’s *t*-test (*p* < 0.05), using Statgraphics Plus 5 software (Statistical Graphics Corp, Rockville, MA, USA).

## 3. Results

### 3.1. Diet and Performance Parameters

The diet presented in Table 1 describes the macro- and micronutrient requirements of pigs, specifically iron, copper, and zinc. At the beginning of the study, no difference in live weight between the control (15.6 ± 2.1 kg) and iron supplemental (14.2 ± 1.7 kg) groups was observed. At the end of the study, no differences were observed for the live weight (44.0 ± 3.6 and 42.0 ± 2.5 kg) and mean weight gain (0.474 ± 0.028 and 0.464 ± 0.046 kg) for control and iron supplemental pigs, respectively.

### 3.2. Hematological Parameters and Serum Micromineral Concentration

At the beginning of the study (day 0), hematological parameters and serum concentrations of iron, copper, and zinc were similar in both groups (Table 3). For hematological parameters, after iron supplementation (day 60), both groups showed an increase in hemoglobin, hematocrit, and transferrin saturation (Table 3). However, the iron supplemental group showed hemoglobin and hematocrit values that were significantly higher than the control group (Table 3). No significant differences were found in serum concentrations of iron and zinc. Serum copper levels increased in both groups as a consequence of time but not as an effect of the treatment (Table 3).

### 3.3. Content of Total Iron, Heme Iron, Copper, and Zinc in Pork

Table 4 shows the total iron, heme iron, copper, and zinc contents after oral iron supplementation (day 60) in the main American pork cuts. Total iron and heme iron contents were similar among the control and iron supplemental groups. Similar to those described for iron, no effects of iron supplementation on copper content in all pork cuts were observed. Importantly, oral iron supplementation had a significant effect of decreasing zinc content in all pork cuts. Zinc content was reduced by 32% in samples of tenderloin and by 55% in sparerib cuts.

### 3.4. Content of Total Iron, Non-Heme Iron, Copper, and Zinc in Pork Offal

Table 5 shows total iron, non-heme iron, zinc, and copper contents in pork offal after supplementation (day 60) from control and iron supplemental groups. The main effect of iron supplementation was an important increase in total iron and non-heme iron contents in most offal, with the exceptions of the heart and brain. The increase in total iron in the pancreas and spleen was close to double, and was 66% for the liver. It was observed that the liver and pancreas almost doubled their non-heme iron content post-supplementation, and the spleen increased its non-heme iron content 3.3-fold. The kidney had a lower increase in non-heme iron just as it had a lower total iron. The heart and brain maintained similar non-heme iron concentrations at the end of the supplementation. Finally, iron supplementation had no effect on the copper and zinc contents in any offal.

## 4. Discussion

The harmful effect of overdoses of orally and parenterally delivered iron on the health of pigs has been extensively studied [14,15,16,17]. However, the effect of high oral iron doses on the content of microminerals in pork cuts has been poorly documented. This issue is very relevant since meat is one of the main sources of iron (especially of heme origin) and zinc for humans [5,6,7].

After oral supplementation with a dose of 3000 ppm iron for 60 days, the pigs showed an increase in some hematological parameters, such as hemoglobin, hematocrit, and transferrin saturation, which is expected in studies of this type and has been described in other studies of oral iron supplementation in pigs of different ages [11,14,26]. The increase in transferrin saturation is explained by the fact that the iron consumed in the diet was absorbed [27]. Approximately 60–70% of body iron is found in the blood as part of erythrocytes, triggering an increase in hemoglobin and hematocrit. This also explains why serum iron did not increase post-supplementation, since excess iron is stored as deposits [27,28] or excreted in urine and feces if iron needs are satisfied [15].

Hepcidin is the main peptide regulator of iron homeostasis in humans and pigs. It has been demonstrated that pigs receiving high doses of iron express greater amounts of hepcidin, which blocks the outflow of iron from enterocytes or macrophages into circulation [16]. This also could explain why no differences in serum iron levels were observed.

Iron supplementation also had no effect on serum copper and zinc concentrations since the diet covered the requirements of both microminerals. Differences in serum levels of copper and zinc following iron supplementation have not been reported [26].

The main effect of high-dose iron supplementation was a considerable reduction in zinc content in all the pork cuts analyzed. In contrast, total iron, heme iron, and copper were found to be within the normal ranges for pork meat (0.5 to 1.2 mg/100 g of iron and 0.08 to 0.15 mg/100 g of copper). Heme iron content fluctuated from 42% to 59% for the control group and from 46% to 60% for the supplemental group, which is considered to be within the normal range for pork meat [7,29,30]. Oral iron supplementation did not affect either total iron or heme iron in meat cuts, which is in agreement with other authors [31,32].

In this study, the zinc content in all pork cuts for the iron supplemental group was below the range considered normal (1.0 to 3.2 mg/100 g) [7,29]. A possible explanation is the antagonistic effect of iron, when consumed in high concentrations, on zinc absorption [33,34], which has been reported in humans [35,36] and pigs [37]. The reduction in zinc absorption may be due to iron-and-zinc-sharing receptors and transporters that participate in their absorption at the small intestinal level, such as the proteins in the ZIP family (ZIP1−14) [38] and the divalent metal transporter 1 (DMT1). Iron reduces zinc entry into DMT1 by up to 50% when the concentrations of these metals are in a 10:1 (iron:zinc) ratio [34]. It has also been observed that hepcidin reduces the expression of DMT1 [35,39], further reducing zinc absorption. Notably, low serum levels of zinc were not found after iron supplementation, but zinc concentrations did decrease dramatically in all pork cuts. This finding could be explained by the fact that muscle tissue is a primary zinc-depositing tissue [27].

Although this was an exploratory study, our findings could contribute to human nutrition because of the high prevalence of zinc deficiency globally [3]. Zinc has been called a “problem” nutrient by the World Health Organization (WHO) because consuming the optimal amount from food is difficult without fortification [40]. However, the experts have indicated that zinc is not necessarily a problem nutrient when meat or some viscera, such as the liver, are consumed regularly [41,42]. As meat is a very important source of zinc, excessive iron supplementation should be seriously considered before implementation as a regular animal husbandry practice. To avoid zinc depletion in pork cuts, it is recommended to follow the iron supplementation recommendations of the National Research Council [22]. Additionally, future studies are needed to establish the maximum doses that can be delivered in iron supplements or in the diet of pigs that do not affect zinc deposition in muscles.

The copper content of pork cuts was similar between groups before and after supplementation, and correspond with ranges previously reported in the literature [7,29]. The copper content of pork was not affected by excessive iron supplementation, possibly because this mineral is primarily captured by the copper transporter enzyme, whereas the DMT1 plays a less important role in this process [34]. Unlike zinc, muscle tissue is not the main site of copper deposition, so it is not a good indicator of copper deficiency [27].

Finally, only iron increased in pork offal after supplementation. This effect was expected for the liver, spleen, and kidney as they are iron reservoir organs [27,43]. Other studies have also reported that iron supplementation increases iron reserves, especially in the liver [15,44]. Fang et al. [45] reported that iron glycine chelate supplementation almost doubled iron concentration in the liver and increased iron concentration by close to 30% in the kidneys. Iron supplementation had no effect on the content of iron in the brain and heart as they are organs that have little relation to iron metabolism.

## 5. Conclusions

Excessive iron supplementation of 3000 ppm decreased the zinc content in pork cuts by 32–55%, whereas total iron, heme iron, and copper contents were not affected. Additionally, supplementation led to the doubling of iron content in the liver, spleen, and pancreas, while the content of zinc and copper in offal remained unaltered. Zinc is a necessary mineral and is most readily consumed via meat. Excess iron supplementation can decrease zinc levels in pork meat cuts, which can negatively impact consumers’ nutrition. Although this study was exploratory with a small sample size, the finding of the dramatic reduction in zinc content in pork cuts due to excessive iron supplementation is important to consider for future studies, as most intensive pork farms perform iron supplementation practices without frequent monitoring.

## Figures and Tables

**Table 1:** Diet composition (as-fed basis).

Ingredient	g/kg
Corn	610.0
Soybean	200.0
Olein	15.0
Sorghum	55.0
Bigolac^®^	20.0
Fish meal	32.0
Wheat bran	50.0
Oyster shell	5.0
Phosbic^®^	3.0
Premix ^a^	3.0
Salkil^®^	2.0
Lysine	3.0
Threonine	2.0
Natuphos^®^	0.5
Salt	0.5
**Composition (%)**	
Crude protein	18.7
Ether extract	6.9
Crude fiber	4.2
Ash	5.8
Free-nitrogen extract	54.5

^a^ Vitamins and minerals (per kg of premix): Vitamin A (9900 UI), Vitamin D (1650 UI), Vitamin E (77 UI), Vitamin K (4.4 mg), choline (330 mg), niacin (44 mg), riboflavin (9.9 mg), B12 (44 mcg), folic acid (770 mcg), biotin (154 mcg), thiamin (3.3 mg), pyridoxine (4.4 mg), Ca (33 mg), Zn (130 g), Mn (45 g), Cu (15 g), I (0.55 g), and Se (0.30 g). The iron content in the control and supplemental groups was 500 and 3000 ppm, respectively.

**Table 2:** Retail names of pork cuts.

Cuts	Retail Names
Shoulder butt	Boston shoulder, pork butt, Boston butt
Picnic shoulder	Shoulder arm picnic, picnic shoulder, fresh picnic, picnic roast
Shoulder hock	Front hock
Center loin	Loin end chop, center loin chop, sirloin chop
Short plate	Short ribs
Spare ribs	Pork ribs, baby back ribs
Tenderloin	Pork filet, pork tender
Ham	Fresh ham, whole ham
Half ham	Shank end, ham hock

**Table 3:** Evolution of hematological parameters and serum concentration of iron (Fe), copper (Cu), and zinc (Zn) of pigs before (day 0) and after iron supplementation (day 60).

Parameters	Day 0			Day 60			Effects (*p*-Value)		
	Fe500	Fe3000	SEM	Fe500	Fe3000	SEM	Treatment	Time	Interaction
Hb (g/dL)	10.9	12.1	0.292	13.6	15.0	0.492	0.006	<0.001	0.176
Ht (%)	35.1	37.3	0.080	40.2	45.2	0.592	0.010	<0.001	0.167
MCV (fL)	53.9	53.8	0.006	54.3	53.4	0.020	0.310	0.432	0.223
Sat (%)	15.7	17.9	0.232	22.8	30.3	0.649	0.218	0.013	0.198
Fe (μg/dL)	85.6	93.0	0.691	116.2	127.9	0.206	0.114	0.132	0.221
Zn (μg/dL)	137.0	139.4	0.255	121.9	122.2	0.018	0.098	0.101	0.296
Cu (μg/dL)	114.0	115.7	0.223	142.2	155.4	0.586	0.172	<0.001	0.098

Control (Fe500) and iron supplemental (Fe3000) groups. Hb: hemoglobin, Ht: hematocrit, MCV: medium corpuscular volume, Sat%: transferrin saturation percentage.

**Table 4:** Effect of oral iron (Fe) supplementation on total Fe, heme Fe, copper (Cu), and zinc (Zn) contents in cuts of pork (mg/100 g).

Cuts	Fe500	Fe3000	SEM	*p*-Value
	Total Fe			
Picnic shoulder	0.7	0.7	0.040	0.769
Spare ribs	0.6	0.6	0.033	0.860
Shoulder hock	0.9	0.9	0.146	0.654
Short plate	0.9	1.0	0.043	0.365
Ham	0.5	0.5	0.092	0.718
Half ham	0.8	0.9	0.048	0.140
Tenderloin	0.9	1.0	0.049	0.331
Shoulder butt	1.0	0.9	0.024	0.331
Center loin	0.5	0.5	0.088	0.810
**Heme Fe**				
Picnic shoulder	0.3	0.3	0.008	0.891
Spare ribs	0.3	0.3	0.047	0.750
Shoulder hock	0.5	0.5	0.008	0.932
Short plate	0.5	0.6	0.037	0.393
Ham	0.2	0.3	0.041	0.143
Half ham	0.5	0.5	0.037	0.111
Tenderloin	0.5	0.5	0.032	0.539
Shoulder butt	0.6	0.5	0.039	0.808
Center loin	0.3	0.2	0.040	0.518
**Cu**				
Picnic shoulder	0.1	0.1	0.014	0.754
Spare ribs	0.1	0.1	0.024	0.212
Shoulder hock	0.1	0.1	0.002	0.692
Short plate	0.1	0.1	0.005	0.005
Ham	0.1	0.1	0.005	0.684
Half ham	0.1	0.1	0.004	0.452
Tenderloin	0.1	0.1	0.019	0.096
Shoulder butt	0.1	0.1	0.008	0.096
Center loin	0.1	0.1	0.019	0.066
**Zn**				
Picnic shoulder	1.8	1.0	0.278	0.001
Spare ribs	2.0	0.9	0.146	<0.001
Shoulder hock	2.5	1.5	0.291	0.002
Short plate	3.0	1.4	0.273	<0.001
Ham	1.5	0.7	0.174	<0.001
Half ham	2.8	1.4	0.122	<0.001
Tenderloin	1.9	1.3	0.206	0.001
Shoulder butt	2.9	1.5	0.196	<0.001
Center loin	1.1	0.6	0.104	<0.001

Control (Fe500) and supplemented (Fe3000) groups.

**Table 5:** Effect of oral iron (Fe) supplementation on total Fe, non-heme Fe, copper (Cu), and zinc (Zn) concentrations in pork offal (mg/100 g).

Offal	Fe500	Fe3000	SEM	*p*-Value
	Total Fe			
Liver	20.6	45.6	6.880	0.003
Spleen	14.9	31.3	2.438	<0.001
Kidney	5.7	7.6	0.362	0.032
Heart	3.8	4.0	0.269	0.666
Brain	1.0	1.1	0.191	0.675
Pancreas	1.3	2.2	0.126	<0.001
**Non-Heme Fe**				
Liver	14.0	32.3	6.688	0.022
Spleen	6.2	20.3	1.834	<0.001
Kidney	3.8	4.5	0.105	0.006
Heart	1.4	1.5	0.227	0.608
Brain	0.6	0.6	0.040	0.480
Pancreas	0.7	1.1	0.050	<0.001
**Cu**				
Liver	0.6	0.6	0.020	0.246
Spleen	0.1	0.1	0.018	0.460
Kidney	0.7	0.6	0.089	0.304
Heart	0.3	0.3	0.019	0.323
Brain	0.3	0.3	0.028	0.825
Pancreas	0.1	0.2	0.037	0.317
**Zn**				
Liver	8.2	8.3	0.425	0.855
Spleen	2.3	2.1	0.060	0.106
Kidney	2.5	2.5	0.140	0.856
Heart	1.6	1.6	0.049	0. 200
Brain	1.1	1.1	0.079	0.728
Pancreas	3.8	4.7	0.771	0.305

Control (Fe500) and supplemental (Fe3000) groups.

## References

[1] Prohaska J.R. (2014). Impact of copper deficiency in humans. Ann. N. Y. Acad. Sci..

[2] Carpenter C., Mahoney A. (1992). Contributions of heme and nonheme iron to human nutrition. Crit. Rev. Food Sci. Nutr..

[3] Hambidge M. (2000). Human zinc deficiency. J. Nutr..

[4] World Health Organization (WHO) (2015). The Global Prevalence of Anaemia in 2011.

[5] Geissler C., Singh M. (2011). Iron, meat and health. Nutrients.

[6] Pereira P., Vicente A. (2013). Meat nutritional composition and nutritive role in the human diet. Meat Sci..

[7] Lombardi-Boccia G., Martinez-Dominguez B., Aguzzi A. (2002). Total heme and non-heme iron in raw and cooked meats. J. Food Sci..

[8] Perri A., Friendship R., Harding J. (2016). An investigation of iron deficiency and anemia in piglets and the effect of iron status at weaning on post-weaning performance. J. Swine Health Prod..

[9] Bowlus C. (2003). The role in T cell development and autoimmunity. Autoimmun. Rev..

[10] Pedersen S., Saeed I., Friis H., Michaelsen K. (2001). Effect of iron deficiency on Trichuris suis and Ascaris suum infections in pigs. Parasitology.

[11] Antileo R., Figueroa J., Valenzuela C. (2016). Characterization of a novel encapsulated oral iron supplement to prevent iron–deficiency anemia in neonatal piglets. J. Anim. Sci..

[12] Churio O., Durán E., Guzmán-Pino S., Valenzuela C. (2019). Use of encapsulation technology to improve the efficiency of an iron oral supplement to prevent anemia in suckling pigs. Animals.

[13] Staroń R., Lipiński P., Lenartowicz M., Bednarz A., Gajowiak A., Smuda E., Krzeptowski W., Pieszka M., Korolonek T., Hamza I. (2017). Dietary hemoglobin rescues young piglets from severe iron deficiency anemia: Duodenal expression profile of genes involved in heme iron absorption. PLoS ONE.

[14] Lipiński P., Starzyński R., Canonne-Hergaux F., Tudek B., Oliński R., Kowalczyk P., Dziaman T., Thibaudeau O., Gralak M., Smuda E. (2010). Benefits and risks of iron supplementation in anemic neonatal pigs. Am. J. Pathol..

[15] Lee S., Shinde P., Choi J., Park M., Ohh S., Kwon I., Chae B. (2008). Effects of dietary iron levels on growth performance, hematological status, liver mineral concentration, fecal microflora, and diarrhea incidence in weanling pigs. Biol. Trace Elem. Res..

[16] Starzyński R., Laarakkers C., Tjalsma H., Swinkels D., Pieszka M., Stys A., Mickiewicz M., Lipiński P. (2013). Iron supplementation in suckling piglets: How to correct iron deficiency anemia without affecting plasma hepcidin levels. PLoS ONE.

[17] Ji P., Lönnerdal B., Kim K., Jinno C. (2019). Iron over supplementation causes hippocampal iron overloading and impairs social novelty recognition in nursing piglets. J. Nutr..

[18] Dove C., Ewan R. (1990). Effect of excess dietary copper, iron or zinc on the tocopherol and selenium status of growing pigs. J. Anim. Sci..

[19] O’donovan P., Pickett R., Plum-Lee M., Beeson W. (1963). Iron toxicity in the young pig. J. Anim. Sci..

[20] Furugouri K. (1972). Effect of elevated dietary levels of iron on iron store in liver, some blood constituents and phosphorus deficiency in young swine. J. Anim. Sci..

[21] National Research Council (NRC) (2005). Committee on Minerals and Toxic Substances in Diets and Water for Animals and Subcommittee on Mineral Toxicity in Animals and Mineral Tolerance of Animals.

[22] National Research Council (NRC) (1998). Nutrient Requeriments of Swine.

[23] Association of Official Analytical Chemists (AOAC) Official Methods of Analysis.

[24] Fischer D., Price D. (1964). A simple serum iron method using the new sensitive chromogen tripiridyl-s-triasine. Clin. Chem..

[25] Rebouche C., Wilcox C., Widness J. (2004). Microanalysis of non-heme iron in animal tissues. J. Biochem. Bioph. Meth..

[26] Rincker M., Clarke S., Einstein R., Link J., Hill G. (2005). Effects of iron supplementation on binding activity of iron regulatory proteins and the subsequent effect on growth performance and indices of hematological and mineral status of young pigs. J. Anim. Sci..

[27] Castellano R., Aguinaga M., Nieto R., Aguilera J., Haro A., Seiquer I. (2013). Changes in body content of iron, copper and zinc in Iberian suckling piglets under different nutritional managements. Anim Feed Sci Tech..

[28] Furugouri K. (1971). Normal values and physiological variations of plasma iron and total iron binding capacity in pigs. J. Anim. Sci..

[29] Olivares M., Pizarro F., Saturnino P., Araya M., Uauy R. (2004). Iron, zinc and copper: Content in common chilean foods and daily intakes in Santiago, Chile. J. Nutr..

[30] Kongkachuichai R., Napatthalung P., Charoensiri R. (2002). Heme and nonheme iron content of animal products commonly consumed in Thailand. J. Food Compos. Anal..

[31] Miller D., Gomez-Basauri J., Smith V., Kanner J., Miller D. (1994). Dietary iron in swine rations affects nonheme iron and TBARS in pork skeletalmuscles. J. Food Sci..

[32] Apple J., Wallis-Phelps W., Maxwell C., Rakes L., Sawyer J., Hutchison S., Fakler T. (2007). Effect of supplemental iron on finishing swine performance, carcass characteristics and pork quality during retail display. J. Anim. Sci..

[33] Lönnerdal B. (2000). Dietary factors influencing zinc absorption. J. Nutr..

[34] Harvey L., Dainty J., Hollands W., Bull V., Hoogewerff J., Foxall R., McAnena L., Strain J., Fairweather-Tait S. (2007). Effect of high-dose iron supplements on fractional zinc absorption and status in pregnant women. Am J Clin Nutr..

[35] Meadows N., Grainger S., Ruse W., Keeling P., Thompson R. (1983). Oral iron and the bioavailability of zinc. Br. Med. J..

[36] Olivares M., Castillo C., Uauy R. (2010). Cobre y cinc. Tratado de Nutrición, Gil., A., Ed..

[37] Hansen S., Trakooljul N., Liu H., Moeser A., Spears J. (2009). Iron transporters are differentially regulated by dietary iron, and modifications are associated with changes in manganese metabolism in young pigs. J Nutr..

[38] Cousins R., Liuzzi J., Lichten L. (2006). Mammalian zinc transport, trafficking, and signals. J. Biol. Chem..

[39] Brasse–Lagnel C., Karim Z., Letteron P., Bekri S., Bado A., Beaumont C. (2011). Intestinal DMT1 cotransporter is down-regulated by hepcidin via proteasome internalization and degradation. Gastroenterology.

[40] Dewey K.G., Brown K.H. (2003). Update on technical issues concerning complementary feeding of young children in developing countries and implications for intervention programs. Food Nutr. Bull..

[41] Hunt J., Gallagher S., Johnson L., Lykken G. (1995). High-versus low-meat diets: Effects on zinc absorption, iron status, and calcium, copper, iron, magnesium, manganese, nitrogen, phosphorus, and zinc balance in postmenopausal women. Am. J. Clin. Nutr..

[42] Hambidge K., Krebs N. (2007). Zinc deficiency: A special challenge. J. Nutr..

[43] Valenzuela C., De Romaña D., Olivares M., Morales M., Pizarro F. (2009). Total iron and heme iron content and their distribution in beef meat and viscera. Biol. Trace Elem. Res..

[44] Yu B., Huang W., Chiou P. (2000). Bioavailability of iron from amino acid complex in weanling pigs. Anim. Feed Sci. Tech..

[45] Fang C., Zhuo Z., Fang S., Yue M., Feng J. (2013). Iron sources on iron status and gene expression of iron related transporters in iron-deficient piglets. Anim. Feed Sci. Tech..

